# Flow-Independent Dark-blood DeLayed Enhancement (FIDDLE): validation of a novel black blood technique for the diagnosis of myocardial infarction

**DOI:** 10.1186/1532-429X-18-S1-O55

**Published:** 2016-01-27

**Authors:** Han W Kim, Wolfgang G Rehwald, David C Wendell, Elizabeth Jenista, Lowie Van Assche, Christoph J Jensen, Enn-Ling Chen, Michele Parker, Raymond Kim

**Affiliations:** 1 Cardiology/Medicine, Duke University Medical Center/Duke Cardiovascular Magnetic Resonance Center, Durham, NC USA; 2 Siemens Health Care Solutions, Chicago, IL USA

## Background

A fundamental component of the CMR exam is contrast enhanced imaging, which is crucial for delineating diseased from normal tissue. Unfortunately, diseased tissue adjacent to vasculature often remains hidden since there is poor contrast between hyperenhanced tissue and bright blood-pool. Conventional black-blood double-IR methods are not a solution; these were not designed to function after contrast administration since they rely on the long native T1 of blood (~2s at 3T) and adequate blood flow within this time period. We introduce a novel **F**low-**I**ndependent **D**ark-blood **D**e**L**ayed **E**nhancement technique (FIDDLE) that allows visualization of tissue contrast-enhancement while suppressing blood-pool signal. We validate FIDDLE in an animal model of myocardial infarction (MI) and demonstrate feasibility in patients.

## Methods

A canine model with variable coronary occlusion times was employed to create a range of MI size/transmurality. Following CMR, hearts were stained with TTC to provide a histopathology reference standard. The main components of FIDDLE are (1) a prep pulse that differentially saturates tissue compared with blood (eg. MT-prep); (2) phase-sensitive IR; and (3) inversion time selection under condition: blood M_Z_ < tissue M_Z_. CMR was performed acutely or chronically at 3T. FIDDLE and delayed-enhancement CMR (DE-CMR) were acquired using matched settings (slice thickness, 7 mm; inplane resolution, 1.2 × 1.0 mm; etc) ~15 minutes after contrast (0.2 mmol/kg). We enrolled patients with enyzmatically confirmed MI and identifiable infarct-related-artery by X-ray angiography, as well as controls with Framingham Risk Score = 0. The patient CMR protocol was similar to that in canines. FIDDLE & DE-CMR analysis were performed separately and masked to identity and pathology (canines) or angiography results (patients).

## Results

**Figure 1:**
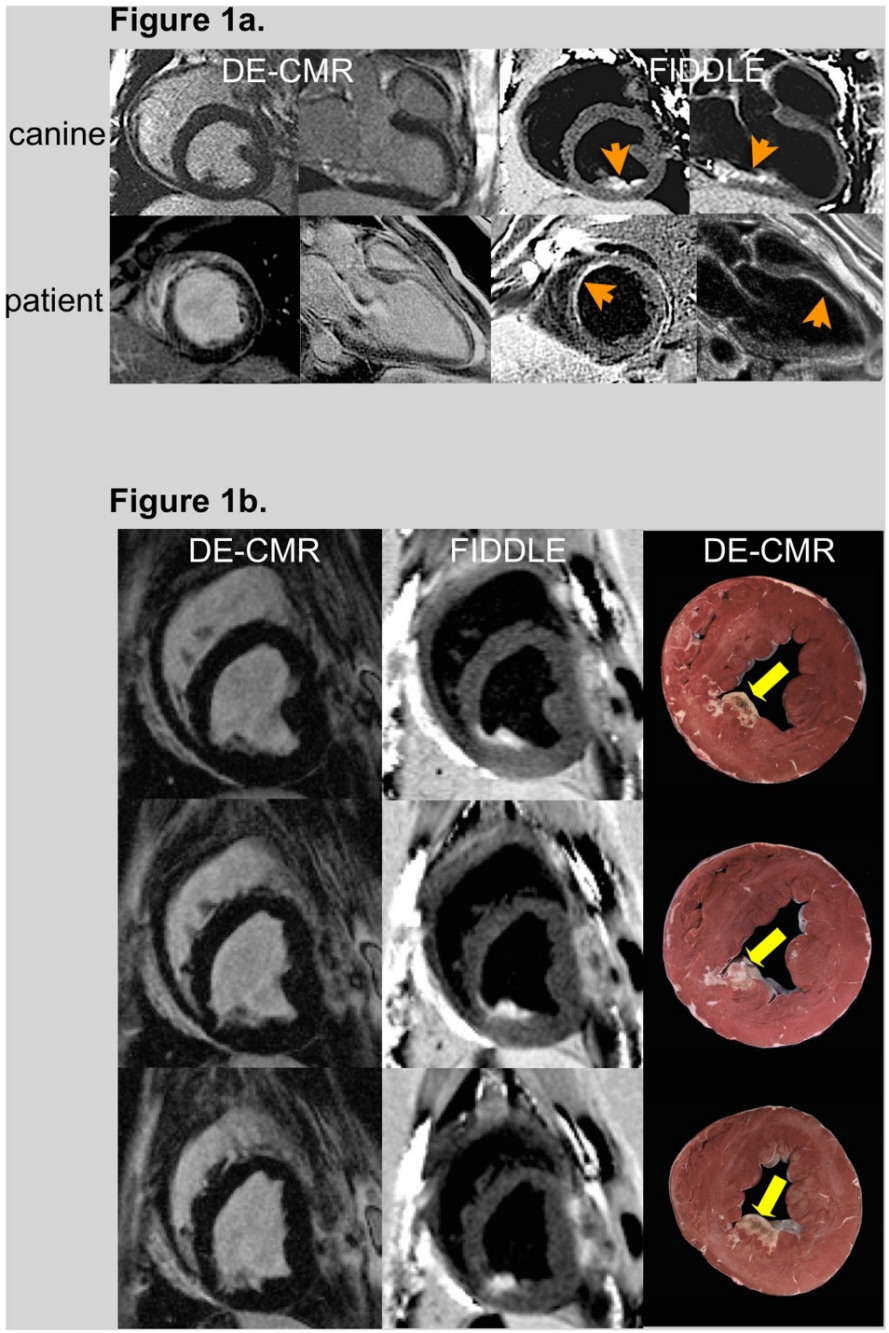
Figure 1

In all canines (n = 22) and patients (MI: n = 20, controls: n = 11), black-blood images were successfully acquired using FIDDLE (Fig 1a). Slow-flow artifacts were not observed on short/long-axis images. Table 1 shows the performance of FIDDLE compared to DE-CMR for the diagnosis of MI in canines (on a slice basis). FIDDLE provided improved sensitivity and accuracy for the detection of MI, particularly in the setting of small, subendocardial infarcts. An example of subendocardial MI detected by FIDDLE but missed by DE-CMR is shown in Fig 1b with pathology reference. The diagnostic performance of FIDDLE was similar in acute and chronic MI. Patient findings were similar in that FIDDLE provided higher accuracy in detecting MI (100% vs 84% for DE-MRI, p = 0.03, on a patient basis).

**Table 1:** Diagnostic Performance in Canines

	Sensitivity	Specificity	Accuracy
Overall			
FIDDLE	97% (95/98)	92% (35/38)	96% (130/136)
DE-CMR	81% (79/98)	95% (36/38)	85% (115/136)
p-value	< 0.001	0.65	0.001
Subendocardial MI (transmurality < 25%)			
FIDDLE	98% (44/45)	92% (35/38)	95% (79/83)
DE-CMR	71% (32/45)	95% (36/38)	82% (68/83)
p-value	< 0.001	0.65	0.008

## Conclusions

We demonstrate that FIDDLE is more sensitive and accurate than standard DE-CMR for the diagnosis of MI. Although validation and feasibility is demonstrated for diagnosis of MI, the technique is easily transferable beyond cardiac imaging and additional applications are expected in other settings (such as vascular wall imaging) where there is need to distinguish abnormal tissue enhancement from blood-pool.

